# Patterns of mental health service utilisation in people with cancer compared with people without cancer: analysis of the Australian National Study of Mental Health and Wellbeing

**DOI:** 10.1007/s11764-023-01472-4

**Published:** 2023-10-04

**Authors:** Huah Shin Ng, Bogda Koczwara, Lisa Beatty

**Affiliations:** 1 Flinders Health and Medical Research Institute, College of Medicine and Public Health, Flinders University, Adelaide, South Australia Australia; 2 SA Pharmacy, Northern Adelaide Local Health Network, Adelaide, South Australia Australia; 3 SA Pharmacy, Southern Adelaide Local Health Network, Adelaide, South Australia Australia; 4 Department of Medical Oncology, Flinders Medical Centre, Adelaide, South Australia Australia; 5 Flinders University Institute for Mental Health and Wellbeing, College of Education, Psychology and Social Work, Flinders University, Adelaide, South Australia Australia

**Keywords:** Cancer, Mental health, Health service utilisation, Australia, Cancer care

## Abstract

**Purpose:**

To compare the patterns of mental health service utilisation between people with and without cancer.

**Methods:**

We performed a cross-sectional study using data of all respondents aged ≥ 25 years from the Australian National Study of Mental Health and Wellbeing 2020–2021 conducted during the COVID-19 pandemic. Comparisons were made between the two groups (cancer versus non-cancer) using logistic regression models.

**Results:**

The study comprised 318 people with cancer (55% female) and 4628 people without cancer (54% female). Cancer survivors had a higher prevalence of reporting poor health (38% versus 16%) and mental distress (18% versus 14%) than people without cancer. There were no significant differences between people with and without cancer in the odds of consulting general practitioner, psychiatrist and other health professionals for mental health, although people with cancer were significantly more likely to consult a psychologist than people without cancer (adjusted odds ratio (aOR) = 1.64, 95%CI = 1.05–2.48). While the odds of being hospitalised for physical health was significantly higher in cancer survivors than people without cancer (aOR = 2.32, 95%CI = 1.78–3.01), there was only a negligible number of people reported being hospitalised for mental health between the two groups. Several factors were associated with higher odds of mental health service utilisation including younger age, unpartnered marital status and presence of a current mental condition.

**Conclusions:**

Alarmingly, despite experiencing higher prevalence of poor health status and mental distress, cancer survivors did not utilise more mental health services than the general population. That is, there is a higher degree of untreated, or undertreated, distress in cancer than in the general population.

**Implications for Cancer Survivors:**

Further research to identify optimal approaches of mental health care delivery for cancer survivors are urgently needed.

##  Introduction

Mental illness is highly prevalent among people diagnosed with cancer with rates ranging from 20 to 53% [1–5]. Despite multiple studies demonstrating the high disease burden of mental illness in cancer, psychosocial issues remain the most frequently reported unmet needs of cancer survivors, including fear of cancer recurrence; future uncertainty; worry about partners, families and friends; help to cope with stress and sexual changes [6]. This may in part be due to mental health stigma, workforce shortages, limited access and affordability of services and prioritisation of physical over mental health [7–9]. Screening and referral of cancer patients for mental distress are not conducted routinely by clinicians despite recommendations of clinical practice guidelines [10]. While several Australian studies have also examined screening [11, 12] and uptake of referrals for psychosocial care [13, 14] in cancer patients, the findings were limited by single-institution experience. Even when services are offered, prior studies have reported low utilisation of mental health services by people with cancer [11, 13, 15, 16]. Further, cancer patients with the highest needs for mental health services appeared to be least likely to be able to afford them [17]. However, given most of these studies were largely conducted in the United States, the generalisability of the findings to other healthcare systems remains to be explored.

We showed in our previous study that Australian cancer survivors utilised health services significantly more than the general population, including outpatient clinics, day admissions and emergency department presentations [18]. Cancer survivors were also more likely to be admitted to hospitals and to consult general practitioners, specialists, nurses and allied health professionals compared with the general population [18]. However, our analysis did not distinguish utilisation rates for mental health services. Mental disorders are frequently reported among people living with cancer in Australia ranging from 16 to 31% [3, 4, 19], but little is known about the patterns and the characteristics associated with healthcare utilisation for mental health in the cancer population. Further, mental well-being of the population as well as the access to health care has been affected by the COVID-19 pandemic [20, 21], but relatively limited data is available to inform the priority for mental health services during a pandemic outbreak.

The Australian National Study of Mental Health and Wellbeing (NSMHW), conducted by the Australian Bureau of Statistics, collected mental health-related information from representative samples of the Australian population to inform mental health policy and development [22]. In the present study, we analysed the most recent NSMHW data conducted during the COVID-19 pandemic to compare (a) patterns of mental health service utilisation and (b) the perceived need for mental health services, between people living with cancer and the general population without cancer. We also examined the characteristics associated with mental health service utilisation.

## Methods

### Data source

We utilised data from the NSMWH 2020–2021 [22]. The data was collected in the survey, which was conducted between December 2020 and July 2021, with 5554 fully responding participants aged 16–85 years living in private dwellings across all Australian states and territories (response rate: 57%) [22]. The de-identified individual-level data was accessed via the Australian Bureau of Statistics DataLab [23].

### Study population

All respondents aged ≥ 25 years who reported having had a history of cancer (whether cancer is current or in remission to reflect the characteristics of the cancer survivorship journey) were identified as the study group of interest (i.e., cancer group refers to ‘people with cancer’), and the remaining participants without a history of cancer were included as the comparison group (i.e., the general population refers to ‘people without cancer’). As the needs for mental health services in adolescent and young adults with cancer (defined as 15–24 years in Australia) [24] are different from adults with cancer, we excluded respondents aged 24 years or younger from the analysis.

### Assessment of services for mental health

Participants were asked whether they had access to any services for mental health. We analysed the type of mental health services accessed in the last 12 months before the interview and/or in their lifetime responded by all participants including: (i) consultations for mental health with any health professionals including general practitioner, psychiatrist, psychologist and other health professionals (mental health nurse, other mental health professionals (e.g., social workers and counsellors), specialist doctor or surgeon (e.g., urologists and cardiologist) and others (e.g., pharmacists and dieticians)); (ii) hospitalisation due to physical or mental health; (iii) digital service use over the phone or using digital technology for mental health including crisis support or counselling services, mental health support groups, forums or chat rooms and the use of treatment programs, training, assessments or other tools to improve mental health.

We also analysed the type of help received for mental health in the last 12 months before the interview including: (i) information about mental illness, its treatment and available services; (ii) medicines or tablets; (iii) counselling (e.g., psychotherapy, cognitive behavioural therapy or counselling); (iv) social interventions (e.g., sought help for housing or money problems or to meet other people for support or company) and (v) skills training (e.g., sought help to look after themselves or their home or to improve ability to work or to use time in other ways). Finally, we examined whether the participants’ perceived needs for mental health services were met for each type of help received for mental health in the last 12 months listed above, and we grouped their responses into two categories (not met/partially met versus met/no need).

Participants were also asked as a separate question, whether they had used any mental health or support services (including general practitioners, psychologist, psychiatrist or other mental health specialist, crisis support or counselling services, online mental health information or mental health apps) during COVID-19 pandemic which began in March 2020.

### Sociodemographic, chronic health condition and self-assessed health/distress co-variables

We extracted the following information on sociodemographic including sex, age, marital status, country of birth, geographical location (measured by remoteness area using the Australian Statistical Geography Standard 2016 [25] and included major cities, inner regional, outer regional, remote and very remote Australia; we combined outer regional, remote and very remote Australia as ‘other areas’ due to small cell sizes), education level, employment status and socioeconomic status (measured by equivalised household weekly income).

Data on current mental health conditions including depression or anxiety reported by the participants as being told by a doctor or nurse that have lasted, or are expected to last, for 6 months or more was extracted. We also calculated the number of other current chronic health conditions including arthritis, osteoporosis, asthma, dementia, diabetes, heart disease, stroke, chronic kidney disease, bronchitis or emphysema and any other long-term health conditions reported by the participants.

Two measures of self-assessed health and distress were extracted for analysis. The first was self-assessed health status that measured perception of health and rated as excellent, very good, good, fair or poor by the participants; we grouped the measurement into two categories (poor health with a rating of fair/poor versus good health with a rating of excellent/very good/good)) [19]. The second was level of mental distress measured by the Kessler Psychological Distress Scale-10 based on a person’s emotional state in the 4 weeks before survey interview; we grouped the measurement into two categories (low–moderate distress level with a score of 10–21 versus high–very high distress level with a score of 22–50)) [19, 22].

### Statistical analysis

The characteristics of the population were summarised using descriptive statistics. Health service utilisation for mental health, the types of help received and the perceived needs of people with and without cancer were compared using logistic regression. The logistic regression analyses were adjusted for sociodemographic characteristics including sex, age, marital status, country of birth, geographical location, education level, employment status and socioeconomic status as well as the number of other long-term chronic health conditions. We also constructed a separate multivariable analysis for the cancer and non-cancer groups to identify characteristics, including sex, age, marital status, country of birth, geographical location, education level, employment status, socioeconomic status, the number of other current long-term chronic health conditions, presence of a current mental health condition, self-assessed health status and level of mental distress, that may be associated with mental health service utilisation in each group. The findings were reported as adjusted odds ratio (aOR) with 95% confidence intervals (CI). Analysis was conducted using R version 4.2.1.

### Ethical consideration

We obtained ethics approval from the Flinders University Human Ethics Low Risk Panel (#6298).

## Results

### Cohort characteristics

The study comprised 318 people with cancer and 4628 people without cancer (Table 1). Relative to people without cancer, people with cancer were older (48% versus 18% aged ≥ 70 years), more likely to be born in Australia (74% versus 67%), live in inner regional/other areas (36% versus 26%), widowed/divorced/separated (38% versus 24%), unemployed (69% versus 36%), have a lower education level (33% versus 27% had no non-school qualification) and a lower socioeconomic status (31% versus 19% in the lowest household income level). Comorbidity was more common in people with cancer (76% versus 51% reported ≥ 1 other health conditions) compared with people without cancer. While the prevalence of mental health conditions was similar between the cancer and non-cancer groups (16% versus 15%), a higher proportion of people with cancer reported a poor health status (38% versus 16%) and a high level of distress (18% versus 14%) than people without cancer.

**Table 1:** Characteristics of the study population

Characteristics, *n* (%)	Cancer, *N* = 381	Non-cancer, *N* = 4628
Sex		
Female	210 (55)	2487 (54)
Male	171 (45)	2141 (46)
Age group in years		
25–39	13 (3)	1324 (29)
40–54	47 (12)	1158 (25)
55–69	139 (36)	1299 (28)
≥ 70	182 (48)	547 (18)
Geographical location		
Major cities	243 (64)	3427 (74)
Inner regional	95 (25)	768 (17)
Other	43 (11)	433 (9)
Registered marital status		
Married	191 (50)	2322 (50)
Widowed/divorce/separated	144 (38)	1103 (24)
Never married	46 (12)	1203 (26)
Country of birth		
Australia	282 (74)	3096 (67)
Others	99 (26)	1532 (33)
Level of highest non-school qualification		
Postgraduate	42 (11)	687 (15)
Bachelor degree	58 (15)	1068 (23)
Diploma	54 (14)	584 (13)
Certificates	101 (27)	1027 (22)
No non-school qualification/level not determined	126 (33)	1262 (27)
Labour force status		
Employed	118 (31)	2943 (64)
Unemployed/ not in labour force	263 (69)	1685 (36)
Equivalised household weekly income		
Decile 1–2 (lowest)	119 (31)	874 (19)
Decile 3–4	103 (27)	836 (18)
Decile 5–6	60 (16)	827 (18)
Decile 7–8	40 (11)	866 (19)
Decile 9–10 (highest)	36 (9)	915 (20)
Not known/not stated	23 (6)	310 (7)
Prevalence of a current mental health condition		
Yes	61 (16)	704 (15)
No	320 (84)	3924 (85)
Number of other long-term health conditions (excl. cancer)		
0	92 (24)	2274 (49)
1	113 (30)	1272 (27)
2	78 (20)	681 (15)
3	56 (15)	264 (6)
4	27 (7)	97 (2)
≥ 5	15 (4)	40 (1)
Self-assessed health status		
Excellent/very good/good	236 (62)	3875 (84)
Fair/poor	145 (38)	753 (16)
Mental distress (grouped Kessler score)		
Low–moderate distress	312 (82)	3983 (86)
High–very high distress	69 (18)	645 (14)

### Health service utilisation for mental health

There were no significant differences between people with and without cancer in the odds of accessing the following services for mental health in the past 12 months: any health professionals, general practitioner, psychiatrist and other health professionals (Table 2). However, people with cancer were significantly more likely to consult a psychologist than people without cancer (aOR = 1.64, 95%CI 1.05–2.48). While the odds of being hospitalised for physical health was significantly higher in people with cancer than people without cancer (aOR=2.32, 95%CI 1.78–3.01), only a negligible number of people reported being hospitalised for mental illness between the two groups. There was also a negligible number of people that used any digital service over phone or digital technology for mental health between the two groups. Similar trends were observed for accessing mental health services in the last 12 months and in their lifetime. No significant differences emerged between people with and without cancer in accessing any mental health or support services during COVID-19 pandemic (aOR = 0.95, 95%CI 0.60–1.43).

**Table 2:** Health service utilisation in the past 12 months and/or in lifetime

Type of health services	Cancer versus non-cancer (reference group) Adjusted odds ratio (95% CI)	
	In the past 12 months *Yes versus no (reference)*	Lifetime *Yes versus no (reference)*
Any services for mental health (hospitalisation or consultations)	0.98 (0.69–1.35)	1.09 (0.86–1.37)
Consultations for mental health:		
(i) Any health professionals^a^	0.94 (0.67–1.31)	1.08 (0.85–1.37)
(ii) General practitioner	0.89 (0.59–1.30)	0.85 (0.65–1.10)
(iii) Psychiatrist	1.14 (0.55–2.13)	0.80 (0.56–1.13)
(iv) Psychologist	1.64 (1.05–2.48)*	1.19 (0.91–1.56)
(v) Other health professionals	0.86 (0.41–1.64)	0.76 (0.48–1.16)
Hospitalisations:		
(i) Physical health	2.32 (1.78–3.01)*	2.43 (1.68–3.61)*
(ii) Mental health	Negligible	0.91 (0.53–1.49)
Digital service use or digital technology for mental health	Negligible	Not applicable (not measured)

The logistic regression models were adjusted for sociodemographic characteristics including sex, age, marital status, country of birth, geographical location, education level, employment status and socioeconomic status as well as the number of chronic conditions

*p-value < 0.05

^a^Any health professionals including general practitioner, psychiatrist, psychologist and other health professionals

### Types of help received for mental health and the perceived needs

There were no significant differences between people with and without cancer in the odds of receiving information about mental illness, medicines or tablets, counselling, social interventions and skills training in the last 12 months (Table 3). There were also no significant differences in their perceived mental health needs between the two groups.

**Table 3:** Type of help received and the perceived needs for mental health

Type of help from hospital/health professional for mental health	Cancer versus non-cancer (reference group) Adjusted odds ratio (95% CI)	
	Whether received help for mental health in the last 12 months *Yes versus no (reference)*	Whether perceived needs were met *Not met/partially met versus met/no need (reference)*
Any mental health services	0.98 (0.69–1.35)	0.74 (0.45–1.15)
Information about mental illness, its treatment and available services	0.89 (0.51–1.45)	0.63 (0.28–1.26)
Medicines or tablets	0.92 (0.59–1.39)	Negligible
Counselling (e.g., psychotherapy, cognitive behaviour therapy or counselling)	1.21 (0.82–1.73)	0.69 (0.34–1.25)
Social interventions (e.g., sought help for housing or money problems or to meet other people for support or company)	1.97 (0.76–4.51)	1.13 (0.53–2.18)
Skills training (e.g., sought help to look after themselves or their home or to improve ability to work or to use time in other ways)	1.56 (0.72–3.05)	0.83 (0.36–1.71)

The logistic regression models were adjusted for sociodemographic characteristics including sex, age, marital status, country of birth, geographical location, education level, employment status and socioeconomic status as well as the number of chronic conditions

*p-value < 0.05 (no findings were significant)

### Factors associated with mental health service utilisation

Four factors were associated with significant differences in accessing services for mental health in the past 12 months (Table 4): age, marital status, education level and the presence of a current mental condition. People who were younger (versus those aged ≥ 70 years), unpartnered (i.e., being widowed/divorced/separated versus being married) and having a current mental condition (versus no current mental condition) were more likely to access health services for mental health than their respective counterparts in both the cancer and non-cancer groups. People with school-level qualification (versus those with a postgraduate qualification) were less likely to access mental health services in both the cancer and non-cancer group. While a higher level of distress (versus low–moderate distress) was also associated with a higher odds of accessing mental health services, these results reached significance for the non-cancer group only, possibly due to a larger sample size in the non-cancer group. Among the non-cancer group, males (versus females) and those who were born overseas (versus born in Australia) had lower odds of accessing mental health services.

**Table 4:** Multivariable analysis identifying characteristics associated with accessing health services for mental health (hospitalisation or consultations in the past 12 months)

Characteristics	Category	Odds ratios (95% CI)	
		Cancer	Non-cancer
Sex	Female	Reference	Reference
	Male	1.79 (0.75–4.43)	0.59 (0.49–0.73)*
Age group in years	25–39	10.93 (1.52–82.45)*	3.54 (2.28–5.55)*
	40–54	3.19 (0.73–14.10)	2.94 (1.92–4.55)*
	55–69	2.71 (1.01–7.46)*	1.87 (1.28–2.76)*
	≥ 70	Reference	Reference
Geographical location	Major cities	Reference	Reference
	Inner regional	1.04 (0.37–2.71)	1.10 (0.85–1.43)
	Other	0.69 (0.15–2.55)	1.03 (0.73–1.45)
Registered marital status	Married	Reference	Reference
	Widowed/divorce/separated	3.56 (1.51–8.81)*	1.66 (1.28–2.15)*
	Never married	0.83 (0.19–3.17)	1.41 (1.11–1.79)*
Country of birth	Australia	Reference	Reference
	Others	1.19 (0.45–2.97)	0.64 (0.51–0.81)*
Level of highest non-school qualification	Postgraduate	Reference	Reference
	Bachelor degree	1.02 (0.26–4.08)	0.83 (0.61–1.13)
	Diploma	0.28 (0.05–1.32)	0.80 (0.56–1.15)
	Certificates	0.77 (0.21–2.88)	0.73 (0.52–1.01)
	No non-school qualification/level not determined	0.23 (0.06–0.91)*	0.53 (0.38–0.74)*
Labour force status	Employed	Reference	Reference
	Unemployed/not in labour force	1.34 (0.46–3.99)	0.97 (0.73–1.29)
Equivalised household weekly income	Not known/not stated	0.31 (0.01–2.83)	1.11 (0.72–1.70)
	Decile 1–2 (lowest)	0.44 (0.09–2.34)	1.09 (0.76–1.57)
	Decile 3–4	0.53 (0.11–2.66)	0.85 (0.60–1.20)
	Decile 5–6	0.41 (0.08–2.06)	0.91 (0.67–1.25)
	Decile 7–8	1.00 (0.22–4.72)	1.02 (0.75–1.38)
	Decile 9–10 (highest)	Reference	Reference
Current mental health condition	No	Reference	Reference
	Yes	22.11 (8.60–62.06)*	13.77 (11.10–17.14)*
Number of other long–term health conditions (excl. cancer)	0	Reference	Reference
	1–2	0.97 (0.36–2.76)	0.84 (0.67–1.05)
	≥ 3	0.74 (0.20–2.71)	1.12 (0.75–1.66)
Self-assessed health status	Excellent/very good/good	Reference	Reference
	Fair/poor	1.81 (0.71–4.68)	1.13 (0.85–1.49)
Mental distress (grouped Kessler score)	Low–moderate distress	Reference	Reference
	High–very high distress	2.20 (0.78–6.20)	2.85 (2.23–3.65)*

*p-value < 0.05

## Discussion

This Australian study provided an overview and comparison of the patterns of mental health service utilisation between cancer survivors and the general population. Of concern, despite a higher level of distress and a higher prevalence of poor health status in people with cancer than people without cancer, we found no significant differences in the mental healthcare utilisation for a range of services between the two groups, with the exception of consultation with a psychologist. This suggests relative underutilisation among people with cancer. The services examined included consultations with health professionals, hospitalisations and digital technology as well as help received for mental health covering information, medicines, counselling, social interventions and skills training. There were also no significant differences between people with and without cancer in their perceived needs for mental health services.

Low access to mental health services among cancer survivors may be explained by several reasons including low perceived needs, affordability, mental health stigma, lack of care coordination and workforce shortage [8, 26]. A qualitative study conducted in Australia showed that although the majority of cancer survivors participated in an online survey were aware of the specific mental health treatment plan subsidised by the Australian Government, less than one in five reported having a mental health treatment plan due to low perceived needs for such services [8]. It is also possible that cancer survivors may prioritise their physical over mental health [27]. Our findings showed that while people with cancer were significantly more likely to be admitted to hospital for physical health conditions than people without cancer, only a negligible number of people reported being admitted for mental health issues in both the cancer and non-cancer groups. Other studies found the main reasons for hospital admissions in cancer patients were directly related to cancer diagnosis or due to symptoms or effects of cancer treatment [28–32]. Although cancer-related hospitalisations were common, representing about 11% of all hospitalisations in Australia in 2019–2020 [33], mental health–related hospitalisations were less frequent accounting for about 1% of all same day hospitalisations and 6% of all overnight hospitalisations [34].

The only service more frequently accessed by people with as opposed to without cancer was psychologists. Access and referrals to psychological support may be comparatively easier for those with cancer than the general public as a number of public psycho-oncology services are provided as part of the hospital services and the Cancer Council counselling services [35]. Clinical practice guidelines and pathways such as ‘clinical pathways for the screening, assessment and management of anxiety and depression in adult cancer patients’ [36] would also have a role; it is plausible that routine distress screening may happen more frequently in the cancer care than in other settings, thus generating more referrals, with psychologists being the standard first port-of-call. In contrast, our study found no differences in the odds of accessing other health professionals for mental health between the cancer and non-cancer groups. Development and implementation of clinical practice guidelines and pathways for referrals to other health professionals for management could be of value to improve access to allied mental health care services targeting people who need them most. In Australia, many of the mental health-related care provided by allied health professionals such as psychologists, social workers and occupational therapists were delivered privately and only partially subsidised by the Australian government [8, 18, 37, 38] such as through the Better Access to Mental Health Care initiative [39, 40], GP Mental Health Treatment Plan or Chronic Disease Management Plan and Team Care Arrangements [41]. Cost barriers may thus deter access to mental health services especially among people who are not eligible for the subsidised schemes.

Both public and self-stigma about mental health may also play a part which can impede engagement and access to mental health care [42]. Individuals may not disclose psychiatric and distress symptoms due to stigma surrounding mental illness. Although routine screening for mental distress among cancer patients is being undertaken in some health services in Australia, it is not done systematically [43]. At present, there is no national data available on the rate of mental health screening for distress in cancer patients in Australia [43]. Several barriers to the implementation of screening for distress in cancer care have been identified including workforce shortage, competing demands and lack of training and support on psychosocial issues [44, 45].

While digital service or technology offers the possibility of improving access to mental health services during the COVID-19 pandemic [46, 47], there were no differences in using any online services or program between people with and without cancer in our study. This may have been the reflection of the age structure of our study population as we excluded adolescent and young adults aged 15–24 who are the typical targeted population for digital health interventions [48]. Other possible explanation includes lack of awareness and adoption of digital health services, limited availability of tools, education and training to support the delivery of services, poor digital literacy and the preference for face-to-face services over digital health engagement [49]. Further research and evaluation are needed to understand how digital interventions can be integrated into the broader mental and health system to facilitate their implementation in the Australian setting [49]. While there were changes being made for eligible people to receive rebates for additional individual mental health sessions under the Better Access Pandemic Support measure [50], our study found no significant difference between people with and without cancer in accessing any mental health or support services during COVID-19 pandemic. It is possible that cancer patients may have experienced disruptions in care during pandemic [51, 52] and may choose to prioritise their safety and physical health by attending to required appointments only [53]. While the current Australian data showed an increased in mental health service use among the general population during pandemic [54], further studies to characterise the changes in mental health services use over time on specific patient populations are needed to provide insight into the impact of COVID-19 for planning and projecting future service needs.

Our study found no differences in various types of help received for mental health including medicines for mental health between the cancer and non-cancer groups and provided further data for cross-national comparisons. A systematic review and meta-analysis showed a considerable variation in the use of antidepressants among cancer patients across different settings and regions [55]. For example, studies conducted in the USA and Netherlands showed that cancer survivors were more likely to report taking psychotropic medicines compared with the general population [56, 57], while the use of antidepressants were less common in studies conducted in Asia [55].

Several characteristics were associated with higher mental health service utilisation, including younger age, unpartnered marital status and the presence of a current mental condition in both cancer and non-cancer groups as observed in our study. The implementation of integrated mental health care model [58] especially for patients with a high need for mental health services [59–61] such as young adults, being widowed/divorce/separated and having a current mental condition and mental distress, may be of value for improving access to care and health outcomes not only in oncology but also in the general population. Our findings also provide further evidence to support the screening and interventions that target select group of patients who may be at risk of underutilising mental health services such as those with a low education level [60].

Our study has several limitations. The data collected in the survey was self-reported and may subject to recall or response bias. While our study population comprised cancer cases (regardless of whether the cancer is current or in remission) to reflect the journey of cancer survivorship, the sample size was modest and we were not able to differentiate the types or stages of cancer or time from cancer diagnosis as the information was not collected in the survey. The study population also restricted to those living in the private dwellings, excluding people residing in special dwellings such as institutions, nursing homes and boarding houses and the homelessness, which may underestimate the prevalence of mental disorders and health service utilisation for mental health. Nonetheless, our findings provide evidence to inform the development of mental health policy and highlight the need for targeted interventions aim at improving access to mental health services among the Australian population living with cancer.

## Conclusion

Alarmingly, despite experiencing higher prevalence of poor health status and mental distress, cancer survivors did not utilise more mental health services than the general population. That is, there is a higher degree of untreated, or undertreated, distress in cancer than in the general population. Further research to identify optimal approaches of mental health care delivery for cancer survivors are urgently needed to overcome the access gap.

## Data Availability

The analysis was performed using the Australian National Study of Mental Health and Wellbeing detailed microdata. As we are not the data custodians, we are not authorised to make the data available. With the appropriate approvals, the data may be accessed through the Australian Bureau of Statistics.
